# Reframing air pollution as a cognitive and socioeconomic risk

**DOI:** 10.1038/s44407-026-00059-4

**Published:** 2026-03-09

**Authors:** Thomas Faherty, Laura-Jayne A. Ellis-Bradford, Helen Onyeaka, Roy M. Harrison, Francis D. Pope

**Affiliations:** 1 School of Geography, Earth and Environmental Sciences, University of Birmingham, Birmingham, United Kingdom; 2 Birmingham Institute of Sustainability and Climate Action, University of Birmingham, Birmingham, United Kingdom; 3 School of Chemical Engineering, University of Birmingham, Birmingham, United Kingdom

**Keywords:** Environmental sciences, Environmental social sciences

## Abstract

Air pollution is a major environmental health risk, yet its cognitive impact remains under-recognised. Evidence links short- and long-term fine particulate matter (PM_2.5_) exposure to reduced cognitive performance and intelligence quotient (IQ). We estimate global PM_2.5_-related IQ losses of 65 billion points, disproportionately affecting low- and lower-middle-income countries. Current air quality standards may not protect neurological health; this threat to global intellect requires nuanced regulation, targeted mitigation, and cross-sectoral policy.

## Introduction

Globally, air pollution is the leading environmental risk factor for human health. It is a known threat to cardiovascular and respiratory disease^1^ and ranked as a Group 1 carcinogen^2^. However, emerging evidence highlights a previously overlooked impact: the degradation of human cognitive potential. Recent research demonstrates a clear and measurable link between exposure to fine particulate matter air pollution and reduced cognitive function^3,4^, with pronounced implications for children’s neurological development^5,6^.

Here, we explore how air pollution impacts neurological health, highlighting the scale of the issue, differences in vulnerability, and whether current air pollution regulation is adequate to protect cognitive health. We reframe air quality not just as an environmental or physical concern but a critical determinant of human intellectual potential. As well as examining the complexities of cognitive dysfunctions linked to air pollution exposure, we also consider the broader societal implications and possible mitigations.

## Toxic air

The World Health Organization (WHO) recognises air pollution as the leading environmental risk factor to global human health, contributing to increased premature mortality and multiple morbidities^7^. Among air pollutants, particulate matter (PM) in the PM_2.5_ size range (particles with diameters ≤2.5 μm) pose the greatest risk to human health. In 2021, an estimated 4.7 million deaths were linked to ambient PM_2.5_ exposure alone^8^. The WHO has established guidelines, recommending that average PM_2.5_ levels should not exceed 15 μgm^−3^ over 24 h and 5 μgm^−3^ annually^9^. These limits are predominantly based on evidence concerning cardiovascular and respiratory health. However, a growing body of evidence also shows an effect of air pollution on brain health and cognitive function^3^.

PM_2.5_ originates from a wide range of anthropogenic (e.g., vehicle emissions, industrial activities, and energy production) and natural (e.g., wind-blown dust) contributors. Importantly, PM_2.5_ may penetrate deep into the body, translocating to the brain and surrounding tissues^10,11^. Across the lifespan, associations exist between exposure to air pollution and changes to brain structure^12^, beginning in foetal development^13^, and continuing through childhood^5^, adulthood^14^, and older age^15^. The persistent impact of air pollution on brain health across the lifespan highlights the significant threat air pollution poses to cognitive function and intelligence.

The economic implications are substantial. Both indirectly through lower productivity compromising the health and earning power of individuals^16,17^, and directly via healthcare costs associated with neurodegenerative illnesses. In 2019, dementia cost economies globally 1.3 trillion US dollars, potentially rising to 2.8 trillion US dollars as early as 2030^18^.

Importantly, while air pollution is a global problem, its impacts are not uniform. Studies suggest that elevated PM air pollution levels are related to unfavourable changes in intelligence, cognition, and educational attainment, with variations across cities and countries due to differences in exposure and local factors. This highlights the differential nature of the problem, where certain populations face greater exposure coupled with fewer resources to mitigate its impacts.

## Health and cognitive impacts

**Fig. 1:**
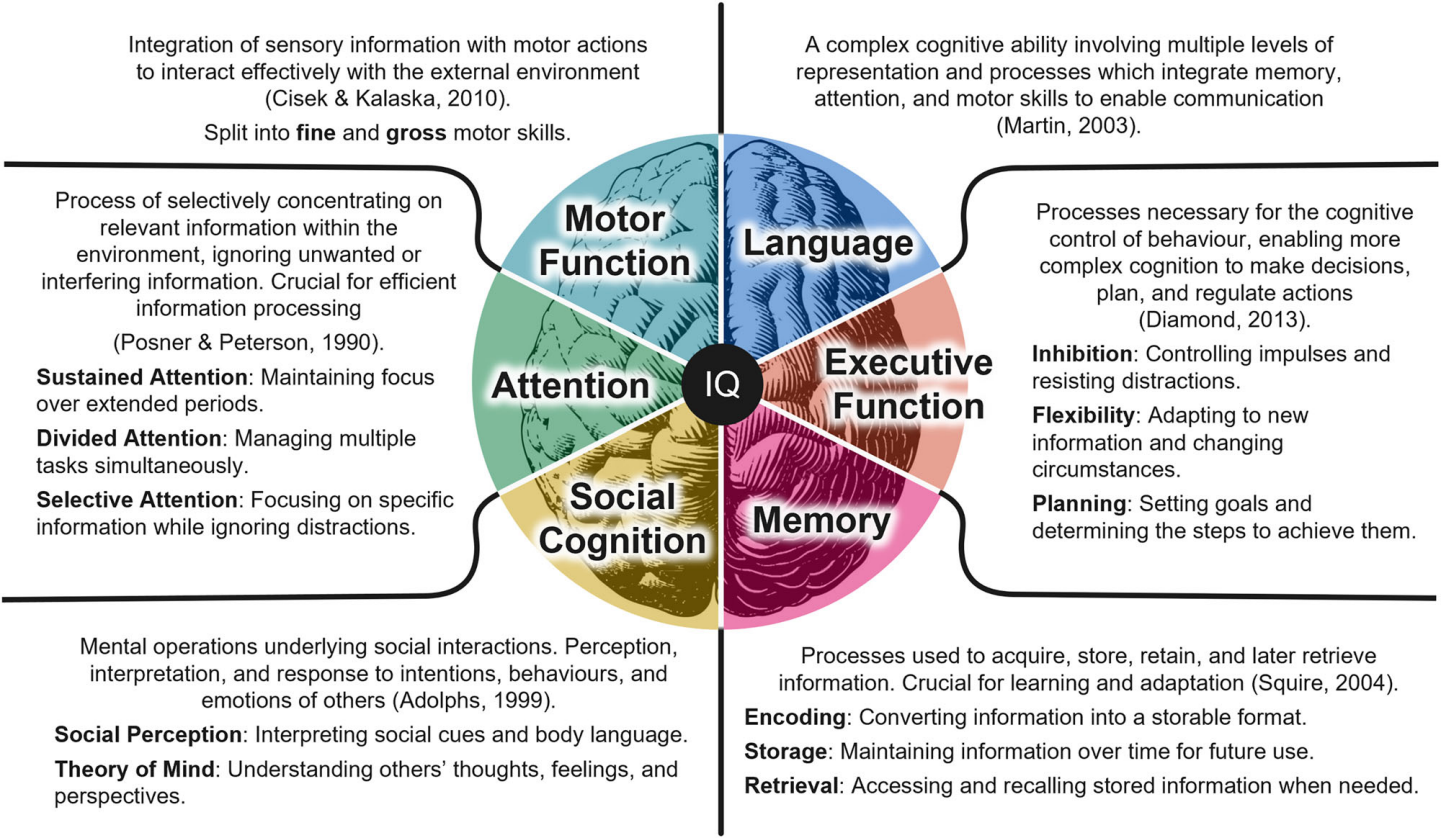
Diagram summarising the key domains within human cognition. Motor Function^60^; Language^61^; Executive Function^62^; Memory^63^; Social Cognition^64^; and Attention^65^.

Emerging research has linked exposure to pollutants such PM_2.5_ to lower Intelligence Quotient (IQ) scores and cognitive dysfunction, particularly in vulnerable groups. Cognition encompasses various facets that function both independently and interdependently to enable successful functioning of an individual. See Fig. 1 for an outline of key cognitive domains of interest; noting that cognitive and functional subdomains span across these. Recent evidence indicates that brief exposure to particulate matter air pollution can temporarily disrupt several of these critical cognitive functions, including attention^19–21^; executive function^21,22^; perceptual-motor function^19,22^; memory^23^; and overall cognitive performance measured by the Mini-Mental State Examination^24,25^.

For the purposes of this paper, we treat IQ as a proxy for overall cognitive function. IQ typically represents an aggregated measure of performance on a battery of tests measuring different cognitive functions. Examples include the Wechsler Adult Intelligence Scale (WAIS), Stanford-Binet Intelligence Scale, and the Universal Nonverbal Intelligence Test (UNIT); which contain subscales such as Working Memory, Perceptual Reasoning (Executive Function), and Processing Speed. Because IQ scores integrate performance across these domains, evidence relating to IQ can reasonably be interpreted as evidence for broader cognitive function.

Studies have consistently shown that children exposed to high levels of air pollution tend to have lower IQ scores, reduced academic performance, and impaired cognitive development. Wang et al.^26^ suggest that an interquartile increase in PM_2.5_ (7.73 μg m^−3^) is associated with a 3.08-point decrease in IQ. In this study the first quartile (2.14–16.08 μg m^−3^) is largely below levels deemed safe by the WHO, but there is limited information regarding differences in cognitive outcomes within this lower range. Lim et al.^27^ provided further evidence by investigating the association between 16-year exposure to PM air pollution and school performance in Denmark. A mean increase of 5 μg m^−3^ in PM_2.5_ was linked to a one-point decrease in GPA (on a 16-point scale), with associations particularly clear for mathematics and natural sciences, similar to studies conducted in the United States^28^.

Alter et al.^29^ conducted a meta-analysis of child (<20 years old) populations with six studies across four countries (China, Italy, Iran, and USA). They estimated a 0.27-point decrease in generalised IQ for every 1 μg m^−3^ increase in PM_2.5_ exposure. These studies assessed multi‑year prenatal or post-natal cumulative exposures while adjusting for key demographic and socioeconomic confounders, including socioeconomic status, maternal IQ, parental education, smoking, and neighbourhood characteristics. Participants ranged from 4 to 11 years old, with one study including a 18–20 year old cohort, and although methodologies varied, every study reported a negative association between PM_2.5_ exposure and IQ, indicating consistent evidence for longer-term developmental impacts. This allows us to forecast future cohort impacts under the assumption that current pollution levels persist.

Our forecast uses a log-linear framework, in contrast to the linear exposure-response relationship adopted by Alter et al. This reflects standard epidemiological practice: long‑term air pollution health models typically assume proportional, not absolute, changes in risk per unit increase in exposure, based on biological reasoning that incremental harm per μg m^−3^ is larger at lower concentrations and diminishes gradually at higher concentrations^30^. Because log‑linear functions also behave almost linearly over narrow concentration ranges, studies often report simple gradients for interpretability while using the log‑linear form for burden estimation. Within the context of neurodevelopment, IQ declines proportionately as pre/post-natal PM_2.5_ increases.

To align with Alter et al.’s linear estimate, we derived the log-linear coefficient (β ≈ 0.00274) by equating the linear and exponential models at the WHO annual PM_2.5_ guideline (5 μg m^−3^)^9^, using 100 as the standardised baseline IQ. This anchors our approach to an internationally recognised reference point while preserving the proportional-effect structure characteristic of air-pollution risk modelling. Applying this parameterisation, our model estimates an IQ loss of ~7.93 points at the mean PM_2.5_ concentration reported in the meta-analysis (30.4 μg m^−3^).

Multiplying this per-person loss by the current global child population aged <15 (2.02 billion^31^) yields an estimated developmental IQ loss of 16 billion IQ points attributable to pre- and post-natal PM_2.5_ exposure. Because all adults alive today were once children, their cognitive development would similarly have followed this concentration-response function had they been exposed to present-day PM_2.5_ levels. Thus, the same framework can be applied to project IQ impacts for future cohorts if PM air pollution concentrations remain unchanged. Assuming a global population of 8.2 billion, the total projected IQ loss rises to ~65 billion points. This estimate is likely conservative because it does not incorporate the effects of later‑life PM_2.5_ exposure on cognitive decline associated with neurodegenerative disease. Current evidence suggests that cognitive impacts of air pollution are likely greatest during two windows: early neurodevelopment and later‑life neurodegenerative decline^32–34^. Our model explicitly captures the developmental pathway but does not quantify the potential additional burden arising during older‑age cognitive deterioration, beyond the scope of this perspective.

The extrapolation from child-focused data to adult populations is not merely a workaround, but a reflection of a broader gap in the evidence base. In the absence of sufficient adult-focused studies, paediatric data offer a valuable proxy for estimating the global cognitive burden of PM_2.5_. While this approach is informative, it also reflects the current uncertainty around factors such as the timing and duration of exposure, the persistence of cognitive effects over time, and the role of potential confounding variables. While PM_2.5_ chemical composition likely modulates toxicity^35,36^, the current global standard for burden estimation is mass concentration. Because compositional data are not routinely available at a global scale, we present our estimate in terms of PM_2.5_ mass concentration, reflecting the present scientific and regulatory standard. Addressing these knowledge gaps through research that includes adult populations across diverse sociodemographic contexts will help refine and validate these estimates.

Interpreting small IQ differences can be challenging, so we contextualise our estimate using established prenatal risk factors. Previous studies suggest that prenatal alcohol exposure is typically associated with IQ reductions in the region of ~3–7 points ^37–41^, and prenatal smoking with losses of ~1–6 points, depending on dose and population subgroup^42^. These effects are therefore larger on average than our PM_2.5_-related estimate, though they fall within a broadly comparable developmental range. Importantly, however, exposure patterns differ substantially: while alcohol use and smoking during pregnancy affect only a subset of the population, air pollution exposure is ubiquitous, meaning even modest individual‑level effects may translate into substantial population‑level implications.

## Global disparities

It is estimated that 9 out of 10 people breathe air containing levels of pollutants above the WHO guidelines^9^. Importantly, while air pollution is a global problem, its impacts are not uniform, with significant variations in concentrations across cities and countries. This highlights the differential nature of the problem, where certain populations face greater exposure coupled with fewer resources to mitigate its impacts.

**Fig. 2:**
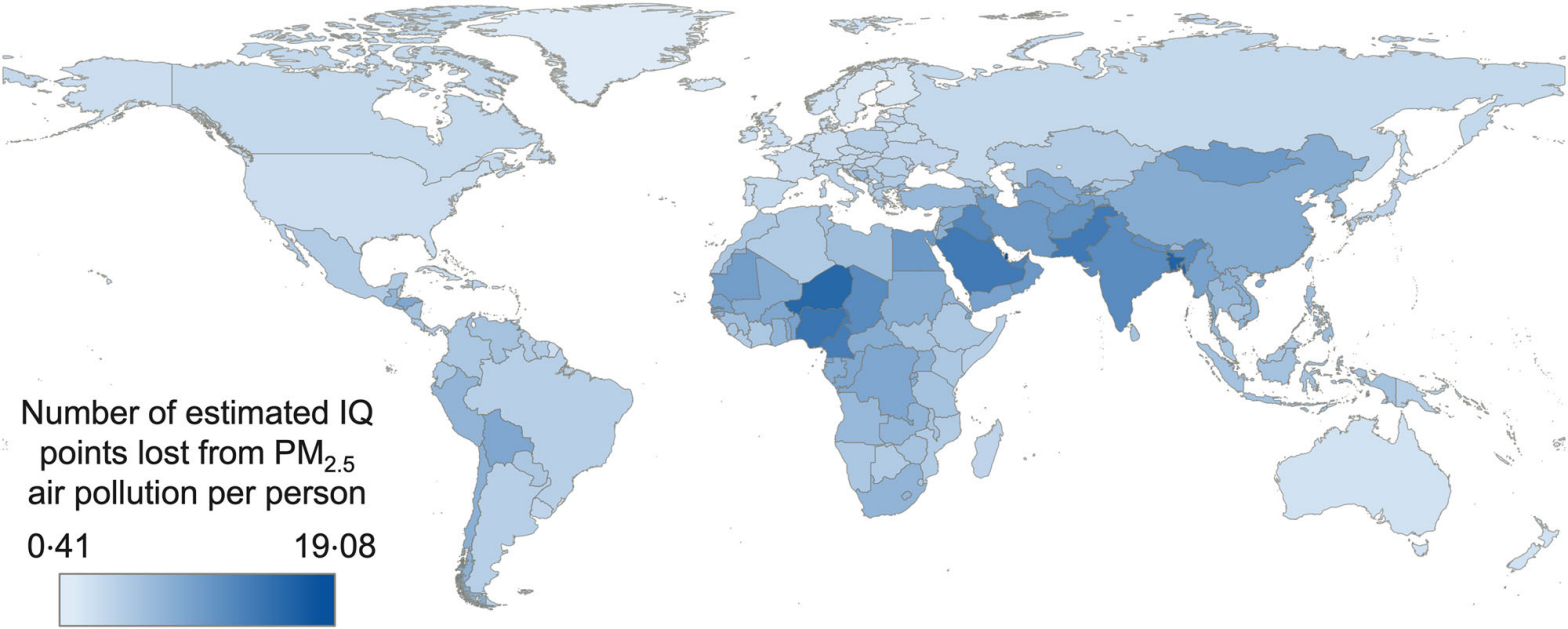
Country-level estimated per person IQ loss attributable to PM_2.5_ exposure during neurodevelopment under 2023 air quality conditions. Population-weighted mean PM_2.5_ concentrations are used. Darker blue shades represent areas with higher estimated IQ loss while lighter shades represent areas with smaller estimated losses. An equal interval classification scheme was used to represent the data distribution across countries. Map created using the Free and Open Source QGIS^66^. Basemap created using the Natural Earth dataset (Made with Natural Earth).

Country-level IQ loss was estimated by applying our log-linear framework to 2023 population-weighted mean PM_2.5_ concentrations, derived from the SatPM_2.5_ V6.GL.02.03 dataset developed by the Atmospheric Composition Analysis Group^43^. These values were then visualised using QGIS software to produce a global map of estimated IQ loss, with losses ranging from 0.41 to 19.08 points. An equal interval classification scheme was used to represent the data distribution across countries. See Fig. 2.

To explore global disparities, we examined the relationship between estimated PM_2.5_-related IQ loss and country income group according to World Bank Data^44^, providing a quantifiable indicator of cognitive potential loss inequality. Indeed, country income status was significantly associated with estimated IQ loss attributable to PM_2.5_ exposure. Specifically, lower-income classifications exhibited substantially greater estimated cognitive impacts compared to higher-income classifications.

**Fig. 3:**
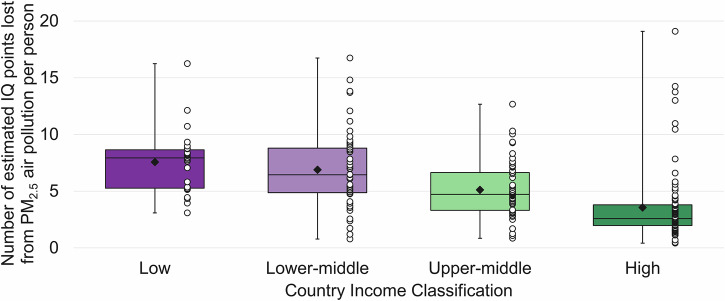
Income-based differences in estimated IQ loss attributable to PM_2.5_ exposure during neurodevelopment under 2023 air quality conditions. Data based on country-level population-weighted mean concentrations across four income categories calculated on Gross National Income per capita^44^. Estimated median IQ loss is represented by horizontal lines, boxes indicate interquartile range (25–75th percentiles), whiskers show full data range between minimum and maximum; white circles indicate individual country data points; black diamonds indicate income classification means.

A one-way independent-measures analysis of variance (ANOVA) was conducted to assess the effect of national income classification (low, lower-middle, upper-middle, and high) on PM_2.5_-related IQ loss. The analysis revealed a statistically significant effect of income group on per-person IQ loss, *F*(3, 211) = 18.700, *p* < 0.001. Post-hoc comparisons, Bonferroni corrected, indicated that the mean IQ loss was significantly different between upper middle-income and all other groups, and high income and all other groups. See Fig. 3.

## Vulnerability and pathways of neurological disruption

Socio-economic factors can exacerbate these outcomes, with deprived communities often suffering the worst effects of pollution due to both their proximity to outdoor pollution sources and increased indoor exposure. Poorer housing conditions, such as inadequate insulation, ventilation, and use of solid fuels, can lead to higher indoor PM_2.5_ levels. The consequences are severe and multifaceted. In the short term, exposure impairs cognitive development in children, leading to poorer academic performance and reduced learning capacity, while in adults it diminishes focus and productivity in the workforce. The economic implications are also significant, as lower IQ levels translate into decreased productivity^45^, higher healthcare costs^46^, and an increased burden on public health systems.

The physical mechanisms driving these cognitive impacts are complex and still debated. Elevated inflammation and oxidative stress in the brain lead to neurodevelopmental disorders. When small enough, particulates can penetrate deep into bodily tissues, translocating to the brain^10^, and causing changes in brain structure and volume^12^, leading to reduced cognitive function^47^.

Within the broader category of particulate air pollution, heavy metals deserve special attention. The cognitive effects of heavy metal exposure are largely attributed to increased neuroinflammation and oxidative stress, which contribute to neurodevelopmental disorders^48^. Lead pollution has long served as a key case study in understanding the cognitive impact of heavy metal pollution exposure. Historically prevalent in the air, lead has been linked to cognitive decline from both short-^49^ and long-term exposure^50^, prompting bans on leaded petrol, for example in the USA (1996) and the EU (2000). Despite significant reductions in airborne lead since the bans, other heavy metals continue to affect cognition yet receive limited attention.

Heavy metal air pollution, particularly from cadmium, chromium, manganese, arsenic, and nickel, has been associated with lower childhood IQ scores and adverse behavioural outcomes^51–53^. Similarly, mercury emissions from industrial processes and combustion have been identified as neurotoxic, with prenatal exposure linked to reduced IQ and developmental disorders^54^. In addition to metal-containing particulate matter, other compositions are likely important including those with high oxidative potential^35^. Moreover, exposure to high levels of air pollution, and short-term exposure during critical developmental periods, also increases the risk of neurodegenerative diseases like Parkinson’s and Alzheimer’s in adulthood^32,55^.

Over the last century, the cognitive ability of the average human has been increasing due to improved nutrition, healthcare and mental stimulation, a trend known as the Flynn effect^56^. However, secular improvements in test performance also mean that age‑related IQ patterns are difficult to track, as the Flynn effect complicates comparisons across cohorts and decades, making pollution‑related cognitive declines later in life particularly challenging to isolate. Despite these measurement complexities, air pollution exposure represents a potential barrier to continued global cognitive gains. The cognitive impacts of air pollution have profound long-term societal consequences, with implications for inter- and intra-country health disparities, especially in a high-tech world that increasingly relies on individuals and societies with superior cognitive capabilities. A critical query emerges: Are the 2021 WHO ‘safe’ limits for PM_2.5_^9^ truly appropriate for cognitive health? The evidence suggests that even modest increases can negatively affect cognitive health, particularly in vulnerable groups such as children. Understanding what happens at these lower levels is essential, as some studies indicate cognitive impacts even below current WHO guidelines.

## Recommendations

Step 1: Policy, regulation, and mitigation

- Governments should enforce stronger air quality standards and curb emissions from vehicles, industries, and power plants.
- Air quality policies must go beyond physical health to also address the neurotoxic impacts of pollution on brain health.
- Urban planning should be designed to minimise exposure, especially in residential areas and around schools, to better protect children’s developing brains^57^.
- Regulatory agencies should set conservative limits that can be revised as new evidence becomes available.
- Standards should be reassessed by considering particle size, chemical composition, and different exposure pathways, rather than focusing on PM_2.5_ levels alone^58^.
- The integrated approach to testing and assessment (IATA), used for the assessment of chemicals, should be adopted to combine epidemiological and toxicological evidence for more robust risk evaluation^59^.

By establishing evidence-based and compositionally relevant regulatory limits, this approach could more effectively mitigate associated health risks. An example approach would be:

1. Set initial exposure limits using existing data from well-studied pollution sources and those known to be chemically similar.
2. Establish conservative limits for unknown sources.
3. Mandate further research into understudied sources and long-term particulate effects.
4. Conduct planned review periods (i.e., 3–5 years), as new research emerges.

This approach reflects the complexity of understanding the far-reaching impact of air pollution on cognitive functioning and the need for adaptive, evidence-based regulatory frameworks.

Step 2: Awareness and future research directions

- Public and professional awareness campaigns are needed to highlight the effects of air pollution on cognitive health, targeting schools, healthcare providers, and organisations working with vulnerable groups.
- Future research should clarify exposure–response relationships between PM and cognitive outcomes through controlled experimental studies.
- Standardised methodologies must be developed to improve consistency and comparability across studies.
- Research should extend beyond WEIRD (western, educated, industrialised, rich, and democratic) populations to capture global diversity in exposure and vulnerability.
- Future studies must consider that cognitive outcomes have distinct features (e.g., developmental sensitivity, life‑course implications) that differ fundamentally from mortality endpoints.
- Greater attention is required to identify specific pollution sources and their distinct neurological impacts.
- Environmental policies should be reframed as a means of protecting cognitive potential as well as physical health, with the added benefit of reducing long-term healthcare costs and fostering intellectual growth.

However, to effectively mitigate this threat to our cognitive capital, we need to go beyond individual efforts. It requires a unified, cross-government response, mandating collaboration between health, education, and environment ministries to develop integrated policies that protect brain health.

## Conclusion

Particulate matter air pollution represents a profound threat to human cognitive development. PM_2.5_ exposure is linked to cognitive dysfunction and IQ loss, especially in children. These effects are measurable and widespread, leading to environmental health inequalities.

Current air quality standards do not reflect what we now know about cognitive health. That needs to change. Policy can move this forward by:

- Ensuring PM_2.5_ limits reflect neurological risks, not just respiratory and cardiovascular risks.
- Focusing upon key exposure locations (e.g., schools, workplaces, and homes).
- Understand the role of pollution-mediated cognitive dysfunction from a health inequality perspective.
- Regulate based on particle composition, not just size, where toxicological effects have been established.
- Fund research into source- and composition-specific toxicity and neurological effects.
- Design and implement adaptive regulatory frameworks that can respond effectively to new evidence.

By prioritising brain health in environmental regulation, we can mitigate pollution-related cognitive decline, enhance workplace productivity, safeguard educational attainment, and contribute to a more equitable and intellectually resilient global society.

## Data Availability

All data used in this study are secondary and publicly available. The country-level air quality data were obtained from the Atmospheric Composition Analysis Group (SatPM_2.5_ V6.GL.02.03 dataset). The dataset can be accessed at: https://sites.wustl.edu/acag/datasets/surface-pm2-5/. Country-level population and income group classifications were obtained from the World Bank Group. The dataset can be accessed at: https://datahelpdesk.worldbank.org/knowledgebase/articles/906519-world-bank-country-and-lending-groups & https://data.worldbank.org/indicator/SP.POP.TOTL. All materials used in the analysis are available upon request.
